# Slide Selection Introduces Sampling-Induced Prediction Uncertainty in Digital Pathology AI: Evidence from Multi-Slide Breast Cancer Cohorts

**DOI:** 10.3390/cancers18162540

**Published:** 2026-08-07

**Authors:** Onur C. Koyun, Yongxin Guo, Hao Lu, Muhammet Fatih Demir, Abbas Alili, Nabil Rahoui, Diana Cardona, Metin N. Gurcan

**Affiliations:** 1 Center for Artificial Intelligence Research, Wake Forest University School of Medicine, Winston-Salem, NC 27101, USA; yongxin.guo@wfusm.edu (Y.G.); hao.lu@advocatehealth.org (H.L.); muhammet.demir@advocatehealth.org (M.F.D.); abbas.alili@advocatehealth.org (A.A.); metin.gurcan@wfusm.edu (M.N.G.); 2 Department of Pathology, Wake Forest University School of Medicine, Winston-Salem, NC 27157, USA; nabil.rahoui@wfusm.edu (N.R.); diana.cardona@advocatehealth.org (D.C.)

**Keywords:** computational pathology, whole-slide imaging, multiple-instance learning, breast cancer, 21-gene recurrence-score surrogate, HER2, tumor heterogeneity, sampling-induced prediction uncertainty

## Abstract

**Background/Objectives:** Digital pathology models increasingly infer molecular phenotypes from hematoxylin and eosin whole-slide images (WSIs), but most assume that one slide adequately represents patient-level tumor biology. We quantified prediction instability caused by slide selection and evaluated multi-WSI aggregation. **Methods:** Six multiple-instance learning architectures were developed in task-specific subsets of TCGA-BRCA (source cohort: 1006 patients and 1065 WSIs) and independently evaluated in 122 patients (400 WSIs) from CPTAC-BRCA. The clinically realistic comparison was random one-slide-per-patient inference versus patient-level aggregation of all available WSIs. Label-conditioned best- and worst-slide analyses were used only as retrospective oracle bounds. The recurrence-risk endpoint was a research-derived 21-gene recurrence-score surrogate calculated from RNA sequencing rather than a clinically reported Oncotype DX result. **Results:** Random single-slide selection yielded AUCs of 0.77–0.86 for recurrence-risk prediction and 0.65–0.77 for HER2 status. Patient-level multi-WSI aggregation yielded AUCs of 0.81–0.89 and 0.71–0.80, respectively. The architecture-controlled AUC improvement from random selection to aggregation ranged from 0.026 to 0.041 for recurrence risk and from 0.036 to 0.073 for HER2. Aggregation also reduced Brier scores by 0.010–0.028 and 0.009–0.106, respectively. The label-conditioned oracle analyses demonstrated wider theoretical performance ranges of 0.46–0.97 and 0.40–0.92, respectively. Slide-selection sensitivity persisted after excluding tumor-evidence-negative and low-tumor-content WSIs. **Conclusions:** Slide selection is a material source of sampling-dependent prediction uncertainty. Patient-level multi-WSI aggregation mitigated selection-dependent degradation across architectures, although validation using routine institutional material and clinically reported molecular assays remains necessary.

## Simple Summary

AI systems that predict molecular characteristics from breast cancer pathology images often analyze only one whole-slide image per patient. However, different slides from the same tumor can contain different tissue regions and may produce different predictions. We evaluated six multiple-instance learning models using TCGA-BRCA for model development and a multi-slide CPTAC-BRCA cohort for independent evaluation. Predictions changed markedly when different slides from the same patient were selected, including near-chance performance under a retrospective worst-slide oracle that is not a clinically deployable procedure. This instability remained after removing slides with little or no detectable tumor. Combining information from all available slides produced higher patient-level discrimination and reduced severe slide-selection failures without changing the model architecture. These results show that slide choice is an important source of sampling-induced prediction uncertainty and support patient-level, multi-slide evaluation for more reliable computational pathology systems.

## 1. Introduction

Artificial intelligence (AI) is increasingly used to infer molecular and prognostic information from hematoxylin and eosin (H&E)-stained whole-slide images (WSIs). In breast cancer, clinically relevant targets include the Oncotype DX recurrence score and human epidermal growth factor receptor 2 (HER2) status. The commercial Oncotype DX assay measures the expression of 21 genes in hormone receptor-positive, HER2-negative disease to estimate recurrence risk and inform adjuvant chemotherapy decisions [1]. HER2 amplification or overexpression is both prognostic and therapeutically actionable [2]. Morphological correlates of these molecular phenotypes may be detectable in routine H&E tissue, motivating histology-based molecular surrogates [3,4,5].

Most WSI models use multiple-instance learning (MIL), in which patches are treated as instances and aggregated into a slide-level representation [6]. This formulation commonly treats one WSI as the unit of inference [7], although a patient may have multiple sections obtained from different blocks, tumor regions, or section depths. Clinical pathology interpretation typically integrates evidence across several slides, and multigene testing is performed on sections selected to represent the invasive tumor [8]. A single-slide prediction may therefore reflect only a partial and potentially unrepresentative observation of patient-level disease biology.

Breast cancers exhibit spatial variation in cellular composition, architecture, grade, proliferation, necrosis, stromal context, and immune infiltration [9,10,11]. Differences between biopsies, blocks, and deeper sections can consequently affect both pathological assessment and molecular testing [12]. Slide-to-slide prediction variability may also reflect preanalytical and technical factors, including fixation, freezing artifacts, section thickness, tissue folds, staining, scanning, and tumor content. We use the term *sampling-induced prediction uncertainty* for the combined variability observed when different valid tissue sections from the same patient are analyzed while the patient-level target remains fixed [13].

Recent studies have predicted breast cancer recurrence-risk or recurrence-assay-related endpoints from H&E WSIs using regression–classification, conventional deep learning, and prototype-guided MIL approaches [14,15,16]. Other work has addressed intra-slide heterogeneity through prototype learning, heterogeneous graphs, and multiplex-detection MIL [17,18,19], while case-level or multi-image studies have proposed hybrid aggregation and graph-based multi-WSI learning [20,21,22]. These studies primarily focus on developing aggregation architectures or improving endpoint performance. In contrast, the present study isolates the effect of choosing one WSI from the same patient while holding the model, patient cohort, and label fixed.

The contribution of the present study is therefore not a new MIL architecture. Instead, we hold the patient cohort, labels, feature encoder, trained model, and model parameters fixed while only changing the WSI used at inference. This controlled design quantifies within-patient slide-selection sensitivity across six MIL families and two molecular endpoints. We then evaluate a minimal architecture-preserving patient-level strategy in which embeddings from all available WSIs are concatenated into a single bag. Because the same procedure is applied to every architecture without retraining or architecture-specific fusion, the comparison isolates the contribution of broader tissue sampling from the choice of MIL backbone.

Using The Cancer Genome Atlas breast cancer cohort (TCGA-BRCA) [23] for model development and the Clinical Proteomic Tumor Analysis Consortium breast cancer cohort (CPTAC-BRCA) [24] for external evaluation, we study a research-derived RNA-sequencing-based 21-gene recurrence-score surrogate and HER2 status. The clinically relevant comparison is between random one-slide-per-patient inference and patient-level multi-WSI aggregation. Label-conditioned best- and worst-slide analyses are included only as retrospective oracle upper and lower bounds; they are analytical stress tests and are not deployable slide-selection procedures. Tumor-content sensitivity analysis and calibration assessment further examine whether the observed effect is explained by poor tumor representation or unreliable probability estimates. This framework systematically quantifies slide-selection vulnerability and tests a practical architecture-compatible mitigation strategy.

## 2. Materials and Methods

### 2.1. Study Cohorts and Experimental Design

This study used two publicly available breast cancer cohorts. The Cancer Genome Atlas breast cancer cohort (TCGA-BRCA) was used for model development, and the Clinical Proteomic Tumor Analysis Consortium breast cancer cohort (CPTAC-BRCA) was used exclusively for external evaluation. Only patients with available hematoxylin and eosin (H&E) whole-slide images (WSIs) and corresponding molecular annotations were included. All analyses were conducted at both slide and patient levels, with strict patient-wise separation enforced throughout to prevent information leakage.

Two tasks were evaluated: classification of the research-derived 21-gene recurrence-score surrogate (abbreviated as ODX in figures and tables) and HER2 status classification. Ground-truth HER2 labels were obtained directly from cohort annotations. TCGA-BRCA data were used for model training and internal validation, while CPTAC-BRCA data were reserved for independent external testing.

Because TCGA-BRCA and CPTAC-BRCA do not contain clinically reported Oncotype DX assay results, the recurrence-risk endpoint was treated as a research-derived 21-gene recurrence-score surrogate. Upper-quartile-normalized STAR–Salmon gene-level RNA-sequencing values were log2-transformed, row-median-centered, and column-standardized within the corresponding cohort before the published recurrence-score formulation was applied [25]. This procedure uses transcriptomic measurements to construct a research endpoint and does not use WSI model predictions. The resulting normalized score should not be interpreted as the commercial clinical assay value.

No patient-level paired commercial Oncotype DX measurements were available; consequently, direct correlation, agreement, or error relative to the commercial assay could not be estimated. Although the research score uses genes and functional groups related to the 21-gene assay, differences in RNA measurement platform, normalization, tissue selection, analytical calibration, and the proprietary clinical workflow may alter both score values and uncertainty. The prevalence-matched normalized cutoff used below is therefore a research classification threshold and is not numerically equivalent to the clinical recurrence-score cutoff.

### 2.2. Tissue Segmentation and Patch Extraction

All WSIs were processed using a standardized preprocessing pipeline. Tissue regions were segmented from the background using color-based thresholding in the HSV color space, followed by morphological refinement filtering to remove small artifacts. Only tissue-containing regions were retained.

Segmented WSIs were tessellated into non-overlapping patches of size 896 × 896 pixels at 20× magnification. Patches containing insufficient tissue content were excluded. This patch size was selected to preserve sufficient histological context while remaining computationally tractable and is consistent with recent pathology foundation model workflows.

No stain normalization or stain-specific color augmentation was applied. The only intensity normalization was the standard ImageNet channel normalization required by the feature encoder, as described in Section 2.4.

### 2.3. Slide-Level Tumor-Content Quality Control

To assess whether slide-selection effects were driven by tumor-evidence-negative or tumor-poor WSIs, we performed a slide-level tumor-content quality-control analysis on the available CPTAC-BRCA WSI pool. Valid tissue patches were identified using the same tissue-segmentation and patch-extraction pipeline used for model inference. An independent ResNet-18 tumor classifier was then applied to each valid tissue patch. For every patch, the predicted class was assigned by the argmax of the classifier outputs; patches assigned to the tumor class were considered tumor-like.

For each WSI, tumor content was estimated as the fraction of valid tissue patches classified as tumor-like. WSIs with 5% or fewer tumor-like patches were classified as low tumor content, matching the complementary passing criterion of more than 5%. Tumor-content QC labels were not used during model training and were applied only for sensitivity analyses in the external CPTAC-BRCA cohort. For the tumor-content sensitivity analysis, the oracle best- and worst-case slide-selection regimes were recomputed after excluding tumor-evidence-negative or insufficient-QC WSIs and after excluding low-tumor-content WSIs. Each filter was applied separately to the original 400-WSI evaluation subset.

### 2.4. Patch-Level Feature Extraction

Patch-level feature representations were extracted using the official MahmoodLab UNI2-h checkpoint, the successor to the published UNI pathology foundation model [26,27]. Each extracted 896 × 896-pixel region was transformed to the encoder’s required 224 × 224-pixel input and normalized using ImageNet channel means and standard deviations. Each patch was encoded once, and the resulting 1536-dimensional latent embedding was cached for downstream modeling. No task-specific fine-tuning, stain normalization, or stain-specific augmentation was applied.

### 2.5. Multiple-Instance Learning Models

We evaluated multiple state-of-the-art multiple-instance learning (MIL) architectures representing diverse aggregation paradigms:

*Attention-Based Multiple-Instance Learning (ABMIL)* [28]. Attention-based MIL aggregates patch-level features using a learnable attention mechanism that assigns importance weights to individual instances within a slide. The slide-level representation is computed as a weighted sum of patch embeddings, enabling the model to focus on morphologically informative regions while suppressing background tissue. This framework provides a simple and interpretable baseline for weakly supervised WSI analysis.

*Dual-Attention MIL Network (DAMLN).* The DAMLN baseline used in this study was a custom transformer-inspired MIL implementation combining standard multi-head self-attention with an efficient Hydra-style attention block [29]. Input features were projected into a latent space and processed by residual attention blocks with layer normalization and dropout. A learned class token was prepended before the final attention layers, and the normalized class-token representation was projected to the task logits.

*Double-Tier Feature Distillation (DTFT)* [30]. DTFT is a weakly supervised framework designed for whole-slide image (WSI) classification that addresses the challenges of limited slides and massive numbers of patch-level instances. It introduces *pseudo-bags* by randomly partitioning instances from each slide into smaller sub-bags, effectively increasing the number of training bags and mitigating overfitting in small cohorts. A two-tier attention-based MIL pipeline is then applied: the first tier processes the pseudo-bags independently using an attention-based MIL model, and distilled features from these pseudo-bags are fed into a second tier to refine bag-level representations.

*Feature Re-Embedding MIL (RRTMIL)* [31]. RRTMIL employs a feature re-embedding strategy in which patch-level embeddings are iteratively transformed before aggregation. Rather than aggregating instances independently, the model updates instance representations via learnable transformations that refine the feature space organization before pooling. This re-embedding process allows the model to emphasize discriminative feature dimensions and suppress irrelevant variation, while remaining agnostic to spatial ordering or explicit neighborhood structure. As a result, RRTMIL enhances slide-level representation learning without assuming spatial adjacency or sequential relationships between patches.

*S4-Based State-Space MIL Baseline (S4Model).* The S4Model baseline was our MIL adaptation of the structured state-space sequence formulation introduced by Gu et al. [32]. It processes long sequences of patch embeddings with linear sequence-length complexity and was included as a scalable state-space aggregation baseline for WSIs with large instance bags.

*Transformer-Based MIL (TransMIL)* [33]. TransMIL applies transformer architectures directly to sets of patch embeddings, leveraging self-attention to model global contextual interactions across a slide. Positional or structural encodings are used to preserve spatial relationships between instances. This framework enables flexible and expressive aggregation of heterogeneous morphological features at the slide level.

All models used the same UNI2-h patch embeddings and produced slide-level predictions. ABMIL, DTFT, RRTMIL, S4Model, and TransMIL followed their published formulations [28,30,31,32,33]; DAMLN was implemented as the custom dual-attention baseline described above.

### 2.6. Datasets

The TCGA-BRCA cohort was used for model development and internal evaluation. A total of 1133 WSIs were initially available. The dataset comprises samples collected across multiple institutions and scanned using diverse slide scanners and acquisition protocols, thereby incorporating substantial site- and scanner-level heterogeneity into the study design. Research-derived 21-gene recurrence-score surrogate values were calculated from normalized mRNA expression data using the previously published formulation. Because clinically validated multigene assay results are not available in TCGA, the surrogate scores are computed from upper-quartile-normalized STAR–Salmon gene-level expression data [25]. Sequencing data were log2-transformed, row-median-centered, and column-standardized prior to applying the published recurrence score equations.

After quality control—excluding cases with incomplete receptor status, missing gene expression data, or slide processing failures—the final analytical cohort comprised 1065 WSIs from 1006 unique patients. Within the HR+/HER2- subgroup (*n* = 516 patients), the high-risk cutoff followed the prevalence-matching procedure described by Howard et al. [25]. Specifically, the threshold was selected so that approximately 15% of HR+/HER2- TCGA patients were classified as high risk, reflecting the reported proportion of patients with a clinical Oncotype DX recurrence score of 26 or higher in the National Cancer Database [34]. Applied to the processed TCGA score distribution, this procedure yielded a fixed normalized threshold of 0.7169 (score range: −2.009 to 2.744), classifying 443 patients as low risk and 73 as high risk. The same threshold was applied to CPTAC-BRCA without retuning.

Patient-level stratification was enforced to prevent leakage between training, validation, and internal-test partitions. Model development was task-specific: recurrence-score-surrogate training was restricted to the 516 HR+/HER2- patients with eligible surrogate labels, whereas HER2 training used the 980 patients with eligible HER2 annotations. Five patient-level cross-validation folds were constructed separately for each task. The complete 1006-patient, 1065-WSI pool therefore describes the source cohort rather than the sample size of either individual prediction task.

The CPTAC-BRCA cohort was used exclusively for independent external evaluation. The full image pool used for slide-level tumor-content quality control contained 653 WSIs. The predictive multi-slide evaluation subset contained 400 WSIs from 122 patients who met the molecular-label, image-processing, and multi-slide eligibility criteria, corresponding to a mean of 3.28 evaluated slides per patient. No CPTAC data were used for model training or hyperparameter tuning. The research-derived recurrence-score surrogate was calculated using the same STAR–Salmon processing and scoring pipeline applied to TCGA-BRCA. This independent cohort enabled cross-cohort assessment of slide-selection sensitivity and patient-level aggregation. The task-specific cohort composition is summarized in Table 1.

### 2.7. Model Training

Models were trained on TCGA-BRCA using slide-level supervision. For patients with multiple WSIs, each slide was treated as an independent training instance, while strict patient-level separation was maintained across training, validation, and test splits. For each model, five-fold cross-validation was performed at the patient level within TCGA to train, validate, and internally test performance. Binary cross-entropy loss was used for HER2 and recurrence-score-surrogate classification.

Because molecular annotations were defined at the patient level, every eligible slide inherited its patient’s label. This is a standard weak-supervision formulation but can introduce section-dependent label noise: a WSI may contain little visible evidence of the patient-level molecular phenotype even when the label is correct at the patient level. Patient-wise partitioning prevented information leakage, but it did not remove this within-patient supervision ambiguity.

All models were trained for 20 epochs with a batch size of 16 using the AdamW optimizer. The learning rate was initialized at $1\times 10^{-4}$ and decayed to $1\times 10^{-5}$ using a cosine annealing schedule. Early stopping was applied based on validation performance with a patience of five epochs. After internal evaluation across the five TCGA folds, the trained models were applied without further tuning to the multi-slide CPTAC-BRCA cohort for external and cross-cohort assessment. Hyperparameters were held constant across tasks, where applicable, to ensure a fair comparison.

### 2.8. Inference Strategies and Heterogeneity Analysis

To assess the effect of intratumoral heterogeneity and slide selection, we evaluated several inference regimes on the CPTAC-BRCA cohort. In the slide-level inference setting, each whole-slide image (WSI) was treated as an independent sample. The model generated predictions for individual slides using their patch features, while slide-level labels were inherited from the corresponding patient. In this regime, the model does not account for the presence of multiple slides from the same patient and evaluates each slide independently.

In the patient-level random-selection setting, one WSI was sampled per patient without using the outcome label. A single prespecified random draw generated with random seed 42 was applied consistently across architectures; variation reported for this condition therefore reflects the five trained fold models rather than repeated slide-selection draws. To characterize the full range of sensitivity, we also evaluated two label-conditioned oracle regimes. For a positive patient, the oracle-best slide was the WSI with the highest predicted probability and the oracle-worst slide was the WSI with the lowest predicted probability; for a negative patient, these definitions were reversed. Equivalently, slides were ordered by the probability assigned to the patient’s true class. These oracle analyses provide retrospective upper and lower bounds and are not deployable selection strategies.

Finally, we evaluated a patient-level aggregation strategy that integrates information across all available slides for a patient. In this setting, patch-level feature embeddings extracted from every WSI belonging to the same patient were concatenated to form a single patient-level bag within the multiple-instance learning (MIL) framework. The model then generated a prediction from this aggregated set of patches, allowing morphological information sampled across multiple tumor regions and tissue sections to jointly contribute to the final patient-level prediction.

The best-case and worst-case regimes provide theoretical performance bounds to quantify sampling-induced variability; however, such oracle selection is not available in real-world clinical workflows. In practice, slide choice is inherently stochastic and operator-dependent. Patient-level aggregation therefore represents one practical strategy for reducing sampling-dependent prediction variability while preserving a patient-level unit of inference. These inference regimes collectively enable systematic comparison of slide-level instability versus patient-level robustness under realistic multi-WSI conditions.

### 2.9. Performance Evaluation

Model performance was assessed using accuracy, area under the receiver operating characteristic curve (AUC), sensitivity, specificity, positive predictive value, negative predictive value, and F1 score. All models produced continuous outputs. For the descriptive threshold-dependent metrics, the prespecified 0.71 classifier-output threshold used in the evaluation pipeline was applied uniformly and was not tuned on CPTAC-BRCA. This probability cutoff is distinct from the normalized RNA-score threshold of 0.7169 used to construct the recurrence-score-surrogate labels. Probabilistic performance was assessed using the Brier score and reliability diagrams. Metrics were computed at the slide or patient level as defined for each analysis. Unless otherwise stated, means and standard deviations summarize the five independently trained cross-validation models evaluated on CPTAC-BRCA.

### 2.10. Statistical Analysis

The patient was treated as the independent unit for patient-level evaluation. Each of the five cross-validated TCGA models is evaluated separately on CPTAC-BRCA, and discrimination results are reported as the mean ± standard deviation across these trained-model replicates. The folds were not treated as independent external cohorts, and no inferential *p* values were calculated from the five fold-level metrics. Architecture-controlled effect sizes were summarized as the difference between the displayed mean metrics for patient-level aggregation and random single-slide inference. For the calibration figures and Brier scores, probabilities were averaged across the five fold models before patient-level calculation.

Label-conditioned oracle-best and oracle-worst results were interpreted as descriptive performance bounds rather than clinically meaningful competing classifiers. Associations between the number of available WSIs and within-patient dispersion of slide-level predicted probabilities were summarized descriptively using Spearman rank correlation. Tumor-content filtering was not used for training or primary cohort construction and was applied only in the predefined CPTAC-BRCA sensitivity analysis.

### 2.11. Software and Computational Environment

Models were implemented in Python 3.10 using PyTorch 2.1.0, CUDA 11.8, and cuDNN 8.7.0. WSI reading and preprocessing used OpenSlide 3.4.1 and openslide-python 1.2.0. Statistical analyses used Python 3.10 with scikit-learn 1.3.1, SciPy 1.11.3, and statsmodels 0.14.0. Training and inference were performed using an NVIDIA A100 GPU with 80 GB of memory (NVIDIA Corporation, Santa Clara, CA, USA).

## 3. Results

### 3.1. Patient-Level Multi-WSI Aggregation Improves the Clinically Relevant Random-Slide Baseline

The primary comparison evaluated random one-slide-per-patient inference against aggregation of all available WSIs while holding the trained architecture and weights fixed. Random single-slide inference yielded recurrence-score-surrogate AUCs of 0.773–0.862, whereas patient-level aggregation yielded AUCs of 0.814–0.888 (Table 2). The corresponding architecture-controlled improvement was 0.026–0.041 AUC. For HER2, random single-slide AUCs were 0.653–0.768 and aggregated AUCs were 0.708–0.804, corresponding to improvements of 0.036–0.073. F1 score also increased for every architecture and task. Because no model parameters changed between the two inference strategies, these results provide an ablation of the contribution of multi-slide sampling independent of the selected MIL architecture.

The largest observed improvement was obtained with S4Model for HER2 prediction (ΔAUC = 0.073), whereas the smallest was obtained with ABMIL for recurrence-score-surrogate prediction (ΔAUC = 0.026). These values are descriptive differences between the displayed five-model means.

### 3.2. Label-Conditioned Oracle Bounds Reveal the Potential Magnitude of Slide-Selection Sensitivity

**Figure 1:**
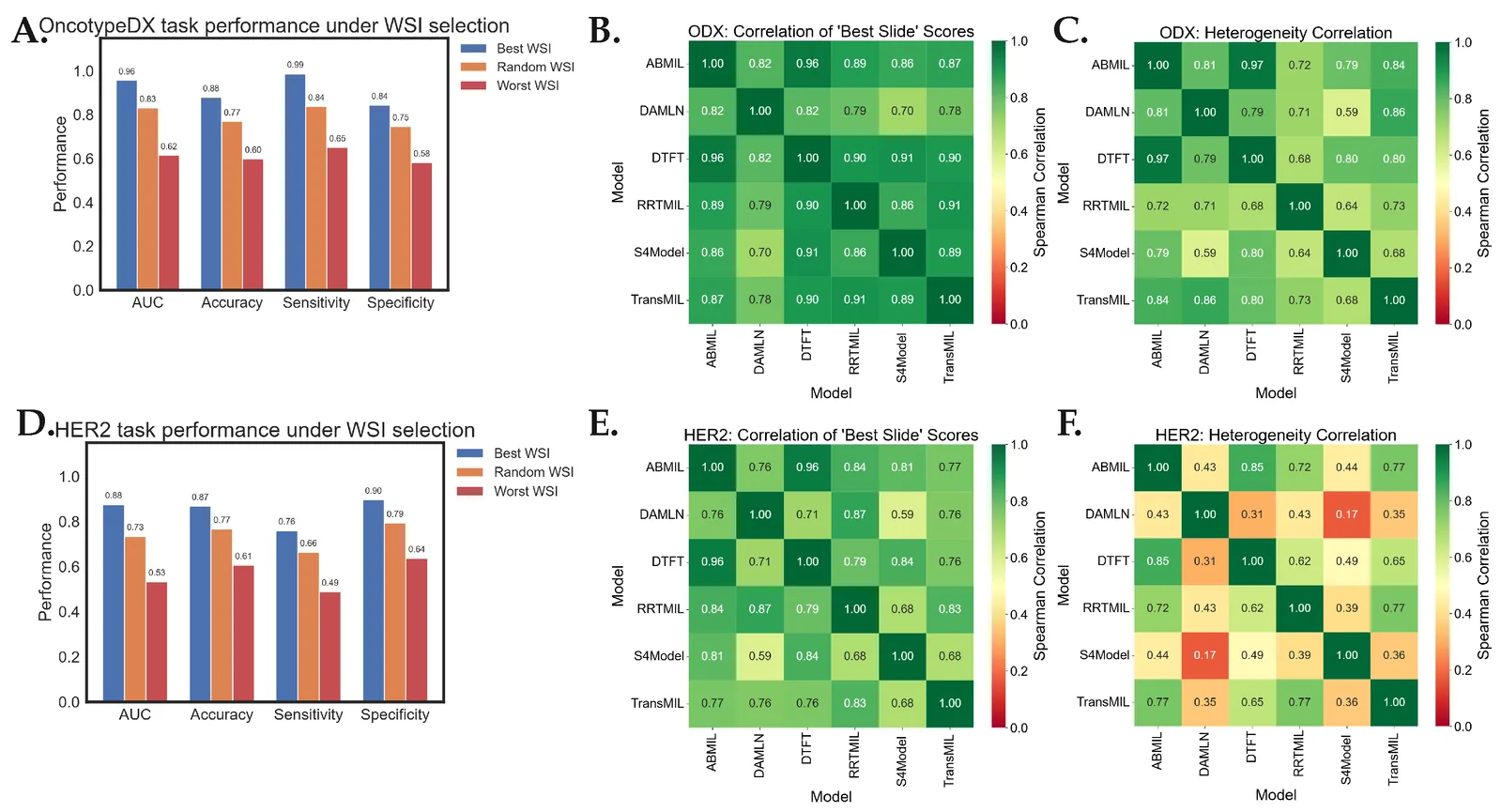
Cross-model performance and concordance under one-slide-per-patient inference. ODX denotes the RNA-derived recurrence-score surrogate rather than a clinically reported Oncotype DX result. (**A**,**D**) Performance averaged across the six MIL architectures for the recurrence-score-surrogate and HER2 tasks, respectively, under oracle-best, random-slide, and oracle-worst selection. Metrics include area under the receiver operating characteristic curve (AUC), accuracy, sensitivity, and specificity. (**B**,**E**) Pairwise Spearman correlations of architecture-specific within-patient slide rankings. For each patient, slides were ordered from oracle-best to oracle-worst using the probability assigned to the patient’s true class: the positive-class probability for positive patients and one minus that probability for negative patients. Higher correlation therefore indicates that different architectures agree on which sections are most and least favorable for the correct patient-level prediction. (**C**,**F**) Pairwise Spearman correlations between model-specific patient heterogeneity scores, defined by the across-slide dispersion of predicted probabilities within each patient. High correlations indicate that different architectures identify similar patients as having pronounced sampling-induced instability.

To bound the full range of slide-selection sensitivity, we next examined label-conditioned oracle-best and oracle-worst selection. Here, the model still produces a slide-level prediction, but the outcome label is used retrospectively to identify the most favorable or least favorable slide for each patient. Using identical patients and fixed model parameters, these procedures produced large changes in apparent patient-level performance across all MIL architectures (Figure 1A–F; Table 3). They are analytical stress tests rather than clinically realizable selection strategies.

**Recurrence-score surrogate (high vs. low risk).** Under best-case selection (most favorable slide per patient), AUCs were consistently high (range: 0.948–0.972; mean = 0.959; Table 3), with F1 scores of 0.745–0.829. Under random selection, performance dropped markedly (AUC of 0.773–0.862; mean = 0.822; Table 3), and F1 decreased to 0.598–0.688. Under worst-case selection, discrimination deteriorated to near- or below-chance levels (AUC 0.460–0.636; mean = 0.561; Table 3), with F1 decreasing to 0.329–0.450. The best-to-worst AUC drop was substantial across all architectures (ΔAUC = 0.33–0.49, depending on the model), demonstrating that slide choice alone can dominate observed performance.

**HER2 (positive vs. negative).** The same pattern held for HER2. Best-case selection yielded strong discrimination (AUC of 0.867–0.919; mean = 0.896; Table 3) with F1 0.640–0.730. Random selection reduced performance (AUC of 0.653–0.768; mean = 0.698; Table 3) and lowered F1 to 0.461–0.545. Worst-case selection again drove performance toward chance or below (AUC of 0.399–0.560; mean = 0.457; Table 3), with F1 of 0.256–0.332. Best-to-worst degradation was again large across models (ΔAUC = 0.35–0.50).

Across all six MIL architectures, the direction of change was consistent from oracle-best to random and from random to oracle-worst selection for both endpoints. The oracle comparisons are reported as effect-size bounds.

### 3.3. Instability Arises Within Patients Under Fixed Cohort Composition

**Figure 2:**
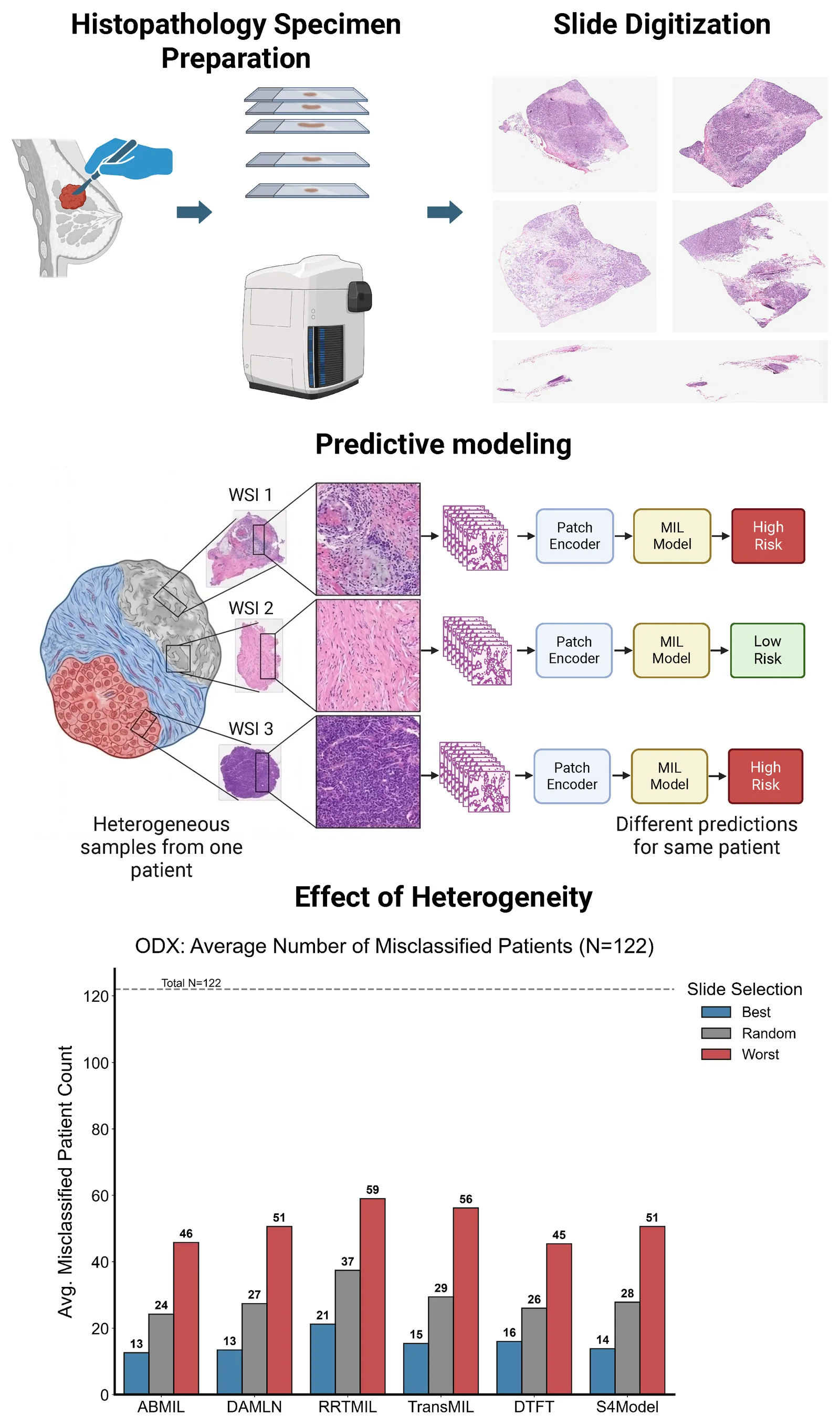
Clinical-to-computational overview of sampling-induced prediction instability. Breast tumor tissue is processed into multiple H&E-stained sections and digitized as whole-slide images (WSIs). Spatially distinct tissue regions from the same patient’s tumor are independently divided into patches, encoded, and analyzed using the same multiple-instance learning model, yet may produce discordant patient-level risk predictions. The histology views are schematic/representative visualizations shown at variable display scales rather than quantitative microscopy panels; the model-processing patches correspond to the study’s 20× extraction setting described in Methods. The lower panel shows the number of misclassified patients in the CPTAC-BRCA cohort (*n* = 122), averaged across the six MIL architectures and five trained fold models, under oracle best-slide, random-slide, and oracle worst-slide selection. Best- and worst-slide selection represent retrospective performance bounds and are not clinically deployable selection strategies.

**Figure 3:**
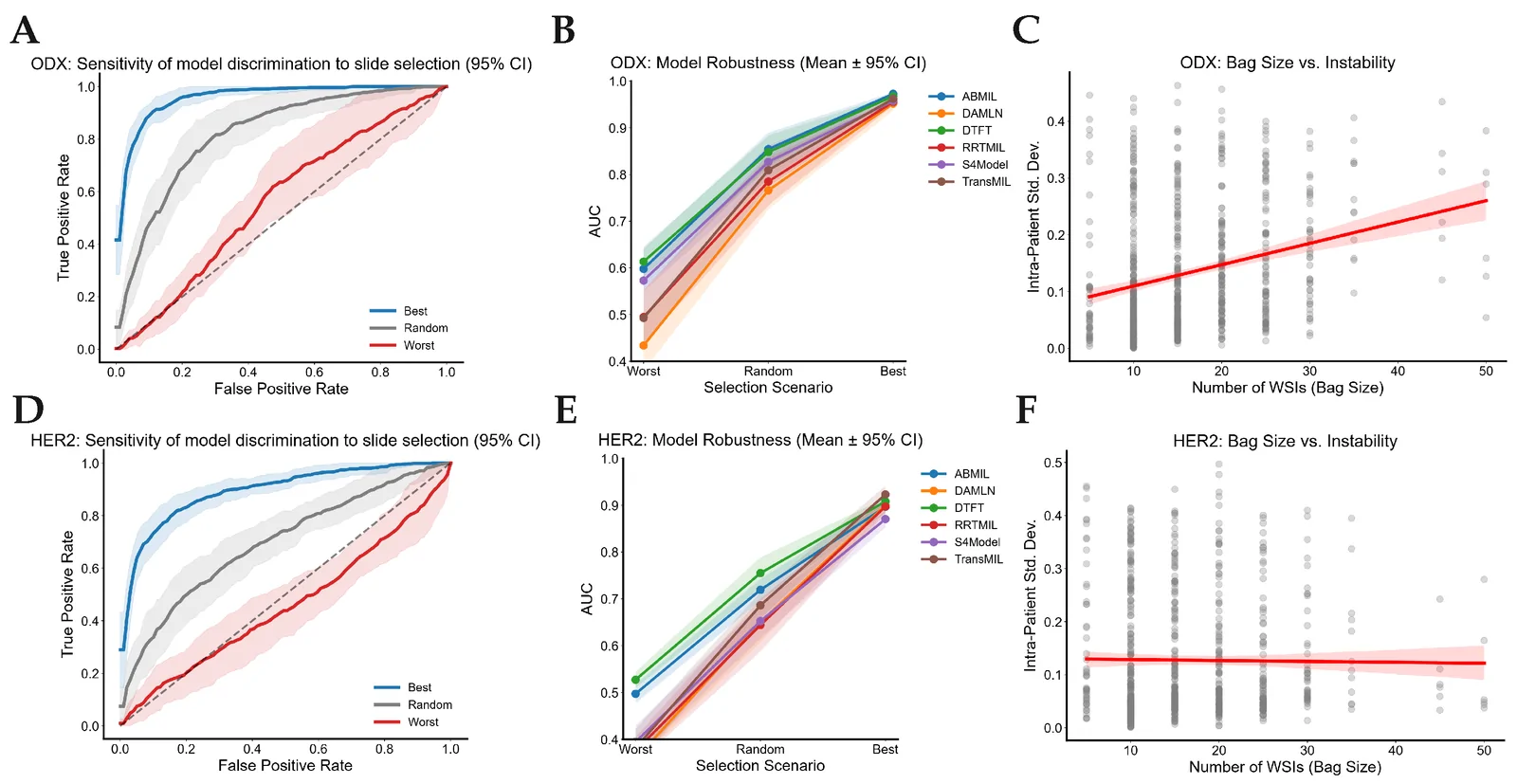
Robustness of recurrence-score-surrogate and HER2 predictions to slide selection and the number of available WSIs. ODX in the figure denotes the RNA-derived recurrence-score surrogate. (**A**,**D**) Receiver operating characteristic curves for the recurrence-score-surrogate and HER2 tasks, respectively, under oracle-best, random-slide, and oracle-worst selection; shaded regions summarize variation across the five trained fold models. The gray dashed diagonal indicates chance-level discrimination. (**B**,**E**) AUC trajectories for each MIL architecture across worst-, random-, and best-slide selection, demonstrating the systematic dependence of apparent model performance on the selected tissue section. (**C**,**F**) Association between the number of available WSIs per patient and the intra-patient standard deviation of slide-level prediction probabilities for the recurrence-score surrogate and HER2, respectively. Each point represents one patient, and the red line shows the fitted trend. Greater within-patient standard deviation indicates greater sampling-induced prediction instability.

Because the inference regimes used the same patients and labels, performance gaps cannot be attributed to changes in cohort composition or outcome prevalence. They instead reflect within-patient, across-slide prediction discordance: different sections from the same patient could produce qualitatively different classifications (Figure 2). Increasing the number of available slides did not uniformly stabilize predictions. For the recurrence-score surrogate, the within-patient standard deviation increased with the number of available slides (Figure 3C); the association was weaker but remained positive for HER2 (Figure 3F). These associations should not be interpreted as evidence that obtaining more slides causes uncertainty. Patients with more available slides simply provide more opportunities to observe biological, sampling, and technical variability.

The present data cannot decompose this discordance into biological intratumoral heterogeneity and preanalytical or technical sources. Differences in sampled block or depth, tumor content, fixation or freezing artifact, tissue folding, section thickness, staining, and scanner characteristics may all contribute. The tumor-content sensitivity analysis below evaluates one component of this variability but does not remove the other potential contributors.

Together, these results separate two distinct notions of “single-slide inference”: (i) slide-level evaluation (all slides treated independently) yields moderate average performance, while (ii) one-slide-per-patient deployment exposes a major failure mode—patient-level performance is highly contingent on slide selection, with best-case and worst-case regimes spanning from near-ceiling to near-chance discrimination across all architectures.

### 3.4. Sensitivity Analysis Excluding Tumor-Evidence-Negative and Low-Tumor-Content WSIs

To determine whether the observed slide-selection instability was driven primarily by tumor-evidence-negative or low-tumor-content WSIs, we performed an additional slide-level tumor-content quality-control analysis in the available CPTAC-BRCA WSI pool. Tumor content was estimated as the fraction of valid tissue patches classified as tumor-like by an independent tumor-detection model, defined as the number of tumor-like patches divided by the number of valid tissue patches.

Across the full CPTAC-BRCA quality-control pool, 653 WSIs were processed. Of these, 648 had valid tissue-patch fractions and were included in the analyzable denominator, whereas five WSIs were excluded because no valid tissue patches were detected. Among the 648 analyzable WSIs, 596 had more than 3% tumor-like patches, 575 had more than 5% tumor-like patches, and 534 had more than 10% tumor-like patches. Automated quality control classified 623 WSIs as likely tumor-present and 17 as likely no- or low-tumor-content; an additional eight WSIs had a low number of valid tissue patches but were retained in the analyzable denominator (Table 4).

The primary multi-slide CPTAC-BRCA evaluation subset contained 400 WSIs. Using ABMIL as the representative sensitivity-analysis model, we repeated the oracle best- and worst-slide analyses after excluding tumor-negative or insufficient-QC WSIs and after excluding WSIs with 5% or fewer tumor-like patches. For recurrence-score-surrogate prediction, the best–worst AUC gap decreased from 0.346 without tumor-content filtering to 0.330 after excluding tumor-negative or insufficient-QC WSIs and to 0.308 after excluding low-tumor-content WSIs. For HER2 prediction, the corresponding gaps were 0.366, 0.318, and 0.267, respectively (Table 4). Although filtering reduced the magnitude of the worst-case degradation, a substantial best–worst gap remained for both tasks.

These findings indicate that tumor-evidence-negative and low-tumor-content WSIs contribute to slide-selection vulnerability but do not fully explain the observed performance gap. Instead, the sensitivity analysis supports the broader interpretation that slide selection introduces uncertainty through multiple mechanisms, including heterogeneous tissue sampling, tumor content, slide quality, and potentially intratumoral biological variation.

### 3.5. Slide-Level Evaluation Provides a Distinct Descriptive Benchmark

When every WSI was evaluated as an independent observation and assigned its patient’s label, recurrence-score-surrogate AUCs ranged from 0.650 to 0.834 and F1 scores from 0.505 to 0.673 across architectures. HER2 slide-level AUCs ranged from 0.521 to 0.652, with F1 scores of 0.289–0.359. These slide-level results use 400 slides as the statistical unit and therefore should not be compared inferentially with patient-level aggregation, which uses 122 patients. They are retained as a descriptive benchmark rather than evidence of an aggregation effect. The paired random-versus-aggregation analysis in Table 2 provides the appropriate architecture-controlled patient-level comparison.

### 3.6. Multi-WSI Modeling Shows Relative Calibration Improvement in Cross-Cohort Evaluation

**Figure 4:**
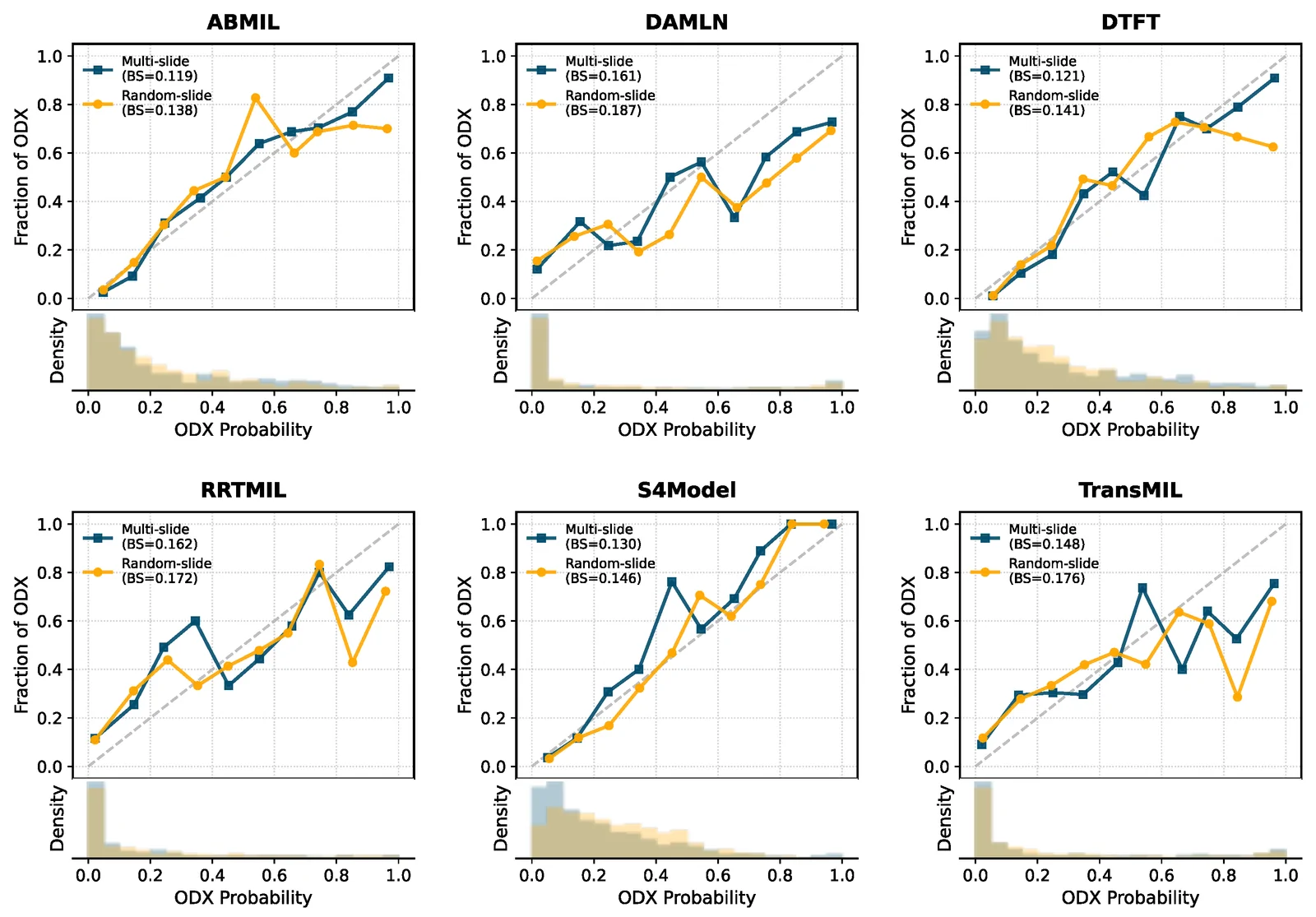
Calibration of RNA-derived recurrence-score-surrogate predictions under multi-slide patient-level aggregation and random single-slide selection. ODX in the figure denotes the research surrogate, not a clinically reported Oncotype DX result. Reliability diagrams are shown for ABMIL, DAMLN, DTFT, RRTMIL, S4Model, and TransMIL. Blue curves represent predictions obtained by aggregating all available WSIs for each patient, whereas orange curves represent predictions obtained from the prespecified random WSI for each patient. The dashed diagonal denotes perfect calibration, where predicted probabilities equal observed outcome frequencies. Histograms below each reliability diagram show the corresponding distributions of predicted probabilities. BS denotes the Brier score, with lower values indicating better probabilistic accuracy. Multi-slide aggregation produced lower Brier scores across all six architectures, with absolute reductions ranging from 0.010 to 0.028.

**Figure 5:**
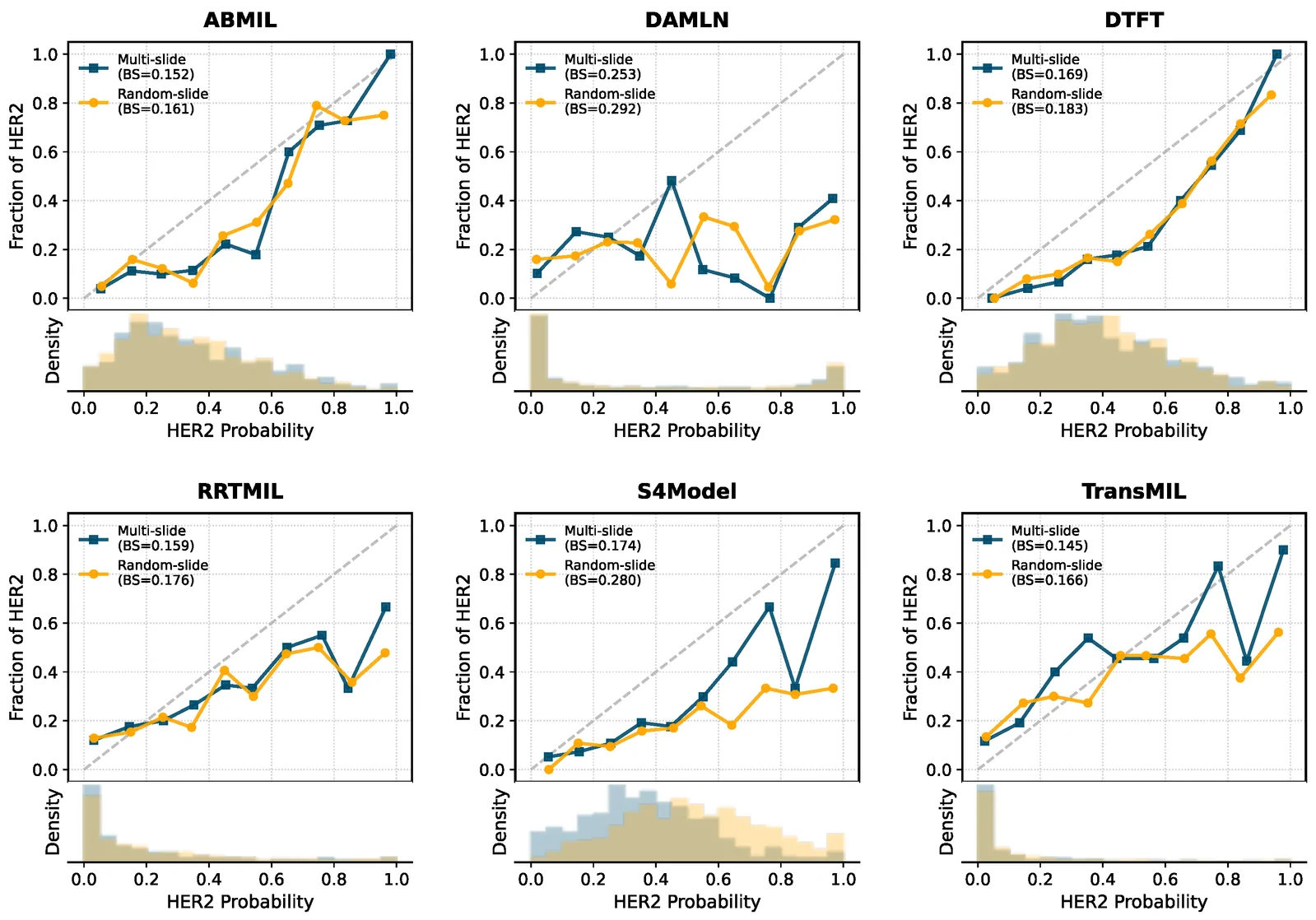
Calibration of HER2 status predictions under multi-slide patient-level aggregation and random single-slide selection. Reliability diagrams are shown for ABMIL, DAMLN, DTFT, RRTMIL, S4Model, and TransMIL. Blue curves represent predictions obtained by aggregating all available WSIs for each patient, whereas orange curves represent predictions obtained from one randomly selected WSI per patient. The dashed diagonal denotes perfect calibration, where predicted probabilities equal observed outcome frequencies. Histograms below each reliability diagram show the corresponding distributions of predicted probabilities. BS denotes the Brier score, with lower values indicating better probabilistic accuracy. Multi-slide aggregation yielded lower Brier scores across all six architectures; reductions ranged from 0.009 to 0.106 and were largest for S4Model.

All models were trained on TCGA-BRCA and evaluated without further tuning on the independent CPTAC-BRCA cohort. Patient-level aggregation lowered the Brier score for every architecture, with absolute reductions of 0.010–0.028 for the recurrence-score surrogate and 0.009–0.106 for HER2 (Table 5; Figure 4 and Figure 5). The largest reduction was observed for S4Model on HER2, for which the Brier score decreased from 0.280 under random single-slide inference to 0.174 after multi-WSI aggregation. The magnitude of improvement varied substantially across architectures, and the reliability curves remained variable, particularly for HER2; these results therefore support relative calibration improvement rather than uniformly well-calibrated predictions.

**Figure 6:**
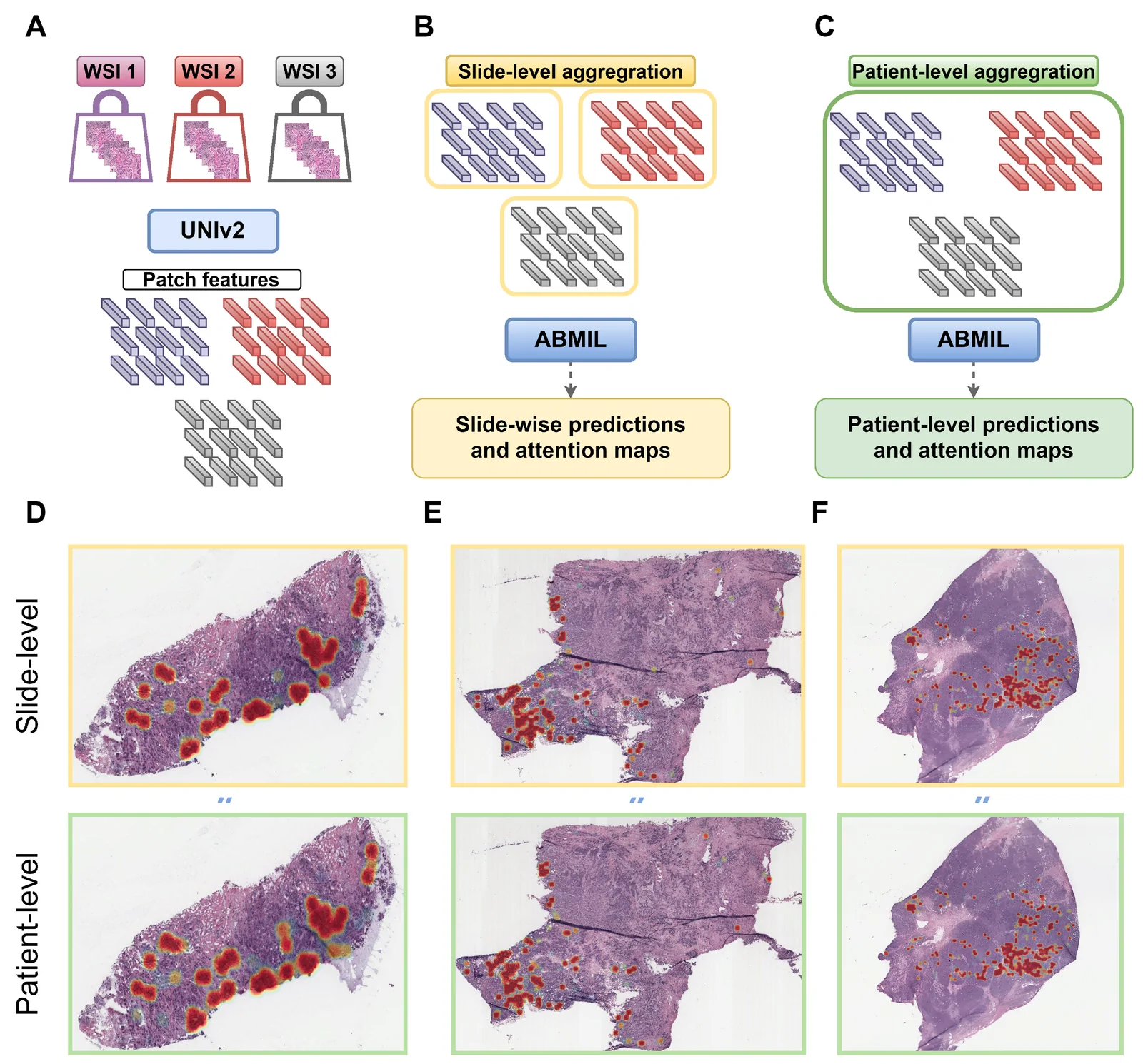
Slide-level versus patient-level multi-WSI aggregation and representative ABMIL attention maps. (**A**) UNI2-h feature extraction from three WSIs belonging to the same patient, with each slide represented by a separate set of patch embeddings. (**B**) In conventional slide-level inference, each WSI is treated as an independent bag and processed separately by ABMIL, producing slide-specific predictions and attention maps. (**C**) In patient-level aggregation, patch embeddings from all available WSIs are combined into a single patient-level bag before ABMIL inference, producing one patient-level prediction while retaining slide-resolved attention information. (**D**–**F**) Matched attention maps for three representative WSIs under slide-level inference (top row; yellow borders) and patient-level aggregation (bottom row; green borders). Warmer attention-overlay colors (yellow/red) indicate relatively higher attention and cooler colors indicate relatively lower attention within each slide-wise visualization. In these representative examples, highly attended regions are visually similar between the two inference strategies; the examples are qualitative and do not establish cohort-wide attention invariance.

We also illustrated how patient-level aggregation affected ABMIL attention maps on three representative CPTAC-BRCA WSIs (Figure 6). In these examples, the spatial locations of highly attended tissue regions were visually similar between slide-level and patient-level inference. Because attention was displayed after slide-wise visualization normalization, the examples should be interpreted qualitatively and do not establish cohort-wide invariance of normalized attention weights. The observed performance improvement is therefore consistent with broader sampling of heterogeneous tumor regions, although quantitative attention-agreement analysis would be required to confirm this mechanism across the cohort.

For standard ABMIL, patch-level attention logits are computed before bag-level normalization; therefore, the relative ranking of patches within an individual slide may remain similar when patches from additional slides are appended. The visual similarity in Figure 6 is thus not independent evidence that the attended regions are biologically meaningful. We use these maps only to illustrate the inference workflow and do not treat attention as a validated explanation. Quantitative cross-architecture attribution analysis and expert pathological annotation remain outside the scope of this prediction-level uncertainty study.

### 3.7. Clinical Implications of Sampling-Induced Instability in Single-Slide Inference

Sampling-induced instability substantially reduced predictive reliability, particularly under the label-conditioned worst-slide oracle. For recurrence-risk prediction, F1 score decreased from 0.745–0.829 under best-slide selection to 0.329–0.450 under worst-slide selection across the six architectures. For HER2 status, F1 score decreased from 0.640–0.730 to 0.256–0.332. These oracle comparisons do not represent deployable slide-selection procedures, but they demonstrate that a less informative slide from the same patient can produce substantial degradation in patient-level classification performance (Table 3).

## 4. Discussion

This study isolates slide choice as a source of prediction variability in histology-based molecular modeling. When one WSI was selected per patient, apparent discrimination depended substantially on the selected section. Patient-level aggregation improved the clinically relevant random-slide baseline for both endpoints across all six MIL architectures, while the label-conditioned oracle analyses demonstrated how large the theoretical selection range could become. The central contribution is therefore a controlled evaluation of sampling sensitivity rather than a new aggregation architecture.

The primary random-versus-aggregation comparison is more relevant to deployment than the oracle analyses. Oracle-best and oracle-worst selection use the true outcome to identify a favorable or unfavorable slide retrospectively and cannot be implemented prospectively. They should be interpreted as stress-test bounds. Realistic workflows could instead use a pathologist’s selection of a representative invasive-tumor block, automated quality control to identify eligible slides, aggregation of all eligible slides, or sequential addition of slides until predictions stabilize. The present results support evaluating such procedures prospectively but do not identify one optimal clinical slide-selection protocol.

Across architectures, ABMIL and DTFT showed the smallest oracle-best–oracle-worst AUC gaps for both tasks, whereas DAMLN, RRTMIL, and TransMIL generally showed larger gaps. The same models did not necessarily achieve the same absolute discrimination, indicating that average performance and selection robustness are related but distinct properties. Simple attention pooling and pseudo-bag distillation may be less sensitive to a localized subset of patches than interaction-dependent or sequence-based representations; however, this is a descriptive pattern across six models and does not establish a causal architectural mechanism. Importantly, no architecture eliminated slide-selection sensitivity, which supports treating tissue sampling as an evaluation-design problem rather than relying on a particular backbone.

The improvement from multi-WSI aggregation was architecture-controlled: the feature encoder, trained MIL weights, patient set, and labels were unchanged, and only the scope of the inference bag differed. This comparison functions as an ablation of broader tissue sampling independent of the MIL backbone. The selected strategy—concatenating patch embeddings from all available WSIs—was intentionally minimal. Alternative methods include mean or confidence-weighted voting over slide predictions, attention-weighted slide fusion, learned fusion networks, hierarchical patch–slide–patient MIL, and heterogeneity-adaptive selection [20,21,22]. Comparing these methods may identify more efficient or robust approaches, but would address a different question from the present controlled analysis.

The term sampling-induced uncertainty should not be interpreted as evidence that all observed variation is biological. Intratumoral heterogeneity provides one plausible mechanism: different sections can contain distinct proportions of invasive tumor, in situ disease, necrosis, stroma, immune infiltrates, and proliferative regions [9,10,11]. However, fixation, fresh-frozen artifact, section thickness, tissue folds, staining intensity, scanner characteristics, block selection, section depth, and tumor content can also change model predictions. The study design measures their combined consequence but cannot decompose their relative contributions. Excluding tumor-evidence-negative and low-tumor-content WSIs reduced, but did not remove, the representative ABMIL oracle gap, indicating that tumor content is contributory rather than sufficient to explain the effect.

Patient-level labels inherited by individual slides create an additional weak-supervision challenge. A molecular label can be valid for the patient while a particular section contains little visible evidence of the corresponding phenotype. Such slides act as conditionally noisy training examples and may attenuate the learned association or encourage reliance on correlates that are inconsistently represented. Strict patient-level data separation prevents leakage but does not resolve this supervision mismatch. Patient-level training or hierarchical objectives may better align label granularity with the available evidence in future work.

The recurrence-risk endpoint requires particularly careful interpretation. This study used a research-derived 21-gene score calculated from RNA sequencing, not a clinically reported commercial Oncotype DX result. No cases had paired commercial assay measurements, so patient-level correlation or agreement with the commercial assay could not be quantified. Differences in RNA platform, normalization, analytical calibration, tissue selection, and assay workflow may introduce endpoint measurement error and may change prediction uncertainty. The prevalence-matched cutoff is likewise not a clinical recurrence-score threshold. Accordingly, the results establish slide-selection sensitivity for an RNA-derived research surrogate and cannot determine performance or uncertainty against actual Oncotype DX testing.

The qualitative ABMIL attention maps illustrate how slide-resolved evidence can be retained after aggregation, but they do not validate biological relevance. Attention values are model-dependent, bag-normalized quantities and should not be equated with causal importance. Moreover, within-slide ABMIL rankings may remain similar by construction when additional instances are appended. Cohort-level attribution stability, perturbation analysis, comparison with alternative explainability methods, and expert annotation of highlighted regions would be needed to support biological claims [35]. We therefore restrict interpretation of Figure 6 to workflow illustration.

Calibration provides complementary information to discrimination because a model may rank patients correctly while producing unreliable probabilities. Multi-WSI aggregation lowered Brier scores relative to random single-slide inference for all evaluated architectures, although the magnitude was architecture- and task-dependent: reductions were modest for ABMIL on HER2 and RRTMIL on the recurrence-score surrogate, but substantially larger for S4Model on HER2. Reliability curves nevertheless remained variable, especially for HER2. These results should therefore be interpreted as relative probabilistic-accuracy comparisons rather than evidence that the models are sufficiently calibrated for clinical use.

Multi-WSI inference also introduces computational costs. Patch encoding grows approximately linearly with the number of processed slides and generally dominates end-to-end computation. Cached embeddings avoid repeated encoder execution, but larger patient-level bags increase storage, data-transfer, memory, and aggregation time. The overhead depends on the MIL architecture: attention pooling can scale approximately linearly in the number of instances, whereas full self-attention may scale quadratically without approximation. Hierarchical pooling, patch subsampling, streaming aggregation, or slide-level fusion could reduce this burden. Formal runtime and memory benchmarking was not performed, so deployment feasibility should be evaluated under the intended hardware and slide-volume constraints.

Several generalizability limitations remain. TCGA-BRCA and CPTAC-BRCA are public research-consortium cohorts and may not reproduce the variation in fixation, staining protocols, scanner vendors, specimen handling, and case mix encountered in community hospitals. CPTAC-BRCA is an independent external cohort, but it is the only external cohort in this study and includes fresh-frozen material that differs from routine formalin-fixed, paraffin-embedded diagnostic tissue. Dataset-specific artifacts or acquisition practices may therefore influence the magnitude of slide-selection variability. Independent institutional and multi-center validation using routine material is required before clinical conclusions are drawn.

Finally, slide-to-slide dispersion may itself be useful as an uncertainty signal. A future workflow could flag patients whose predictions change materially across available slides for additional slide review, pathologist assessment, or molecular confirmation. Such a rule would require a prespecified variability measure, threshold selection using development data, calibration in an independent cohort, and prospective evaluation of coverage and missed-event rates. The current study motivates this uncertainty-aware direction but does not validate a clinical triage policy.

An additional limitation is that the random single-slide condition used one prespecified label-independent draw per trained model rather than repeated Monte Carlo selection. Consequently, its reported standard deviation reflects variation across trained fold models and does not isolate the full distribution of slide-selection randomness. Repeated selection should be incorporated in future validation.

Overall, standard single-WSI benchmarking can conceal a patient-level failure mode: performance may depend on which valid tissue section is analyzed. The random single-slide condition approximates a routine one-slide workflow, oracle selection provides analytical bounds, and multi-WSI aggregation offers a practical architecture-compatible mitigation. Robustness to slide choice should therefore be evaluated alongside discrimination and calibration when histology models target patient-level molecular phenotypes.

## 5. Conclusions

Slide selection materially altered patient-level recurrence-score-surrogate and HER2 predictions across six MIL architectures in an independent multi-slide breast cancer cohort. Patient-level aggregation improved the realistic random single-slide baseline without changing the feature encoder or MIL architectures. The wider label-conditioned oracle range persisted in the representative ABMIL sensitivity analysis after tumor-negative, insufficient-QC, and low-tumor-content WSIs were excluded, indicating that tumor content contributes to, but does not fully explain, selection vulnerability.

These findings support patient-level evaluation whenever multiple WSIs are available and establish slide choice as an important component of model uncertainty assessment. The results do not constitute validation against commercial Oncotype DX testing. Validation using routine formalin-fixed, paraffin-embedded institutional material; clinically reported molecular assays; prespecified slide-selection procedures; and prospective uncertainty-aware workflows is required before clinical deployment.

## Figures and Tables

**Table 1:** Task-specific cohort composition. Counts are reported at the patient level unless otherwise specified. Positive and negative denote HER2 status; high-risk and low-risk denote the recurrence-score-surrogate classes.

Cohort	Task	Patients	WSIs	Positive/High-Risk	Negative/Low-Risk	Mean WSIs/Patient
TCGA-BRCA	Recurrence-score surrogate	516	545	73	443	1.06
TCGA-BRCA	HER2 status	980	1040	154	826	1.06
CPTAC-BRCA	Recurrence-score surrogate	122	400	31	91	3.28
CPTAC-BRCA	HER2 status	122	400	25	97	3.28

**Table 2:** Architecture-controlled comparison of random one-slide-per-patient inference and patient-level multi-WSI aggregation on CPTAC-BRCA. Values show mean ± standard deviation across the five trained fold models. ΔAUC is calculated as aggregated minus random AUC using the displayed means. The random condition uses the prespecified seed-42 slide draw described in Section 2.8.

Task	Model	Random AUC	Aggregated AUC	ΔAUC	Random F1	Aggregated F1
ODX surrogate	DTFT	0.854 ± 0.038	0.885 ± 0.009	0.031	0.677 ± 0.046	0.697 ± 0.026
	ABMIL	0.862 ± 0.039	0.888 ± 0.016	0.026	0.688 ± 0.029	0.701 ± 0.018
	S4Model	0.828 ± 0.038	0.868 ± 0.014	0.040	0.645 ± 0.048	0.674 ± 0.033
	DAMLN	0.773 ± 0.048	0.814 ± 0.064	0.041	0.627 ± 0.041	0.664 ± 0.061
	RRTMIL	0.797 ± 0.055	0.830 ± 0.022	0.033	0.598 ± 0.060	0.654 ± 0.030
	TransMIL	0.815 ± 0.037	0.848 ± 0.041	0.033	0.638 ± 0.049	0.650 ± 0.033
HER2	DTFT	0.768 ± 0.033	0.804 ± 0.009	0.036	0.545 ± 0.053	0.557 ± 0.024
	ABMIL	0.734 ± 0.025	0.775 ± 0.015	0.041	0.535 ± 0.041	0.586 ± 0.043
	S4Model	0.661 ± 0.040	0.734 ± 0.021	0.073	0.461 ± 0.040	0.499 ± 0.027
	DAMLN	0.667 ± 0.044	0.733 ± 0.029	0.066	0.464 ± 0.066	0.506 ± 0.041
	RRTMIL	0.653 ± 0.065	0.708 ± 0.042	0.055	0.466 ± 0.050	0.481 ± 0.041
	TransMIL	0.703 ± 0.034	0.740 ± 0.041	0.037	0.474 ± 0.044	0.540 ± 0.044

**Table 3:** One-slide-per-patient sensitivity to slide selection on CPTAC-BRCA. Oracle-best and oracle-worst regimes use outcome labels retrospectively to bound sampling-induced uncertainty and are not deployable procedures. Random selection approximates the clinically realistic single-slide setting and uses the prespecified seed-42 slide draw. Values show mean ± standard deviation across five trained fold models.

Task	Model	Best AUC	Best F1	Random AUC	Random F1	Worst AUC	Worst F1	ΔAUC
ODX surrogate	DTFT	0.966 ± 0.003	0.791 ± 0.041	0.854 ± 0.038	0.677 ± 0.046	0.636 ± 0.034	0.450 ± 0.094	0.330
	ABMIL	0.972 ± 0.004	0.829 ± 0.030	0.862 ± 0.039	0.688 ± 0.029	0.626 ± 0.047	0.427 ± 0.039	0.346
	S4Model	0.953 ± 0.010	0.806 ± 0.064	0.828 ± 0.038	0.645 ± 0.048	0.593 ± 0.026	0.405 ± 0.063	0.360
	DAMLN	0.948 ± 0.017	0.805 ± 0.048	0.773 ± 0.048	0.627 ± 0.041	0.460 ± 0.064	0.329 ± 0.051	0.488
	RRTMIL	0.956 ± 0.008	0.745 ± 0.068	0.797 ± 0.055	0.598 ± 0.060	0.528 ± 0.066	0.408 ± 0.036	0.428
	TransMIL	0.959 ± 0.009	0.793 ± 0.063	0.815 ± 0.037	0.638 ± 0.049	0.523 ± 0.072	0.355 ± 0.085	0.436
HER2	DTFT	0.909 ± 0.008	0.710 ± 0.048	0.768 ± 0.033	0.545 ± 0.053	0.560 ± 0.017	0.332 ± 0.018	0.349
	ABMIL	0.896 ± 0.016	0.712 ± 0.052	0.734 ± 0.025	0.535 ± 0.041	0.530 ± 0.024	0.328 ± 0.041	0.366
	S4Model	0.867 ± 0.021	0.640 ± 0.106	0.661 ± 0.040	0.461 ± 0.040	0.420 ± 0.041	0.268 ± 0.020	0.447
	DAMLN	0.897 ± 0.017	0.644 ± 0.042	0.667 ± 0.044	0.464 ± 0.066	0.399 ± 0.013	0.256 ± 0.022	0.498
	RRTMIL	0.888 ± 0.019	0.671 ± 0.076	0.653 ± 0.065	0.466 ± 0.050	0.407 ± 0.042	0.263 ± 0.036	0.481
	TransMIL	0.919 ± 0.023	0.730 ± 0.076	0.703 ± 0.034	0.474 ± 0.044	0.426 ± 0.047	0.258 ± 0.047	0.493

**Table 4:** Tumor-content quality control in the full CPTAC-BRCA WSI pool and ABMIL sensitivity analysis in the multi-slide CPTAC-BRCA evaluation subset. Tumor-like fraction was defined as the number of tumor-like patches divided by the number of valid tissue patches. Panel A summarizes slide-level quality control across all processed WSIs. Percentages use the 648 analyzable WSIs as the denominator. Panel B reports mean ABMIL AUCs across the five trained folds and the corresponding oracle best–worst one-slide-per-patient gap after separately applying each filter to the original 400-WSI evaluation subset.

**A. Slide-Level Tumor-Content Quality Control**					
**Criterion**		**Number of WSIs**		**Percentage of Analyzable WSIs**	
Total WSIs processed		653		–	
Analyzable WSIs with valid tissue-patch fractions		648		100.00%	
>3% tumor-like patches		596		91.98%	
>5% tumor-like patches		575		88.73%	
>10% tumor-like patches		534		82.41%	
Likely tumor-present by automated QC status		623		96.14%	
Likely no- or low-tumor-content by automated QC status		17		2.62%	
Low valid-tissue-patch WSIs (retained in analyzable denominator)		8		1.23%	
Excluded—no valid tissue patches detected		5		–	
**B. ABMIL Sensitivity Analysis of Best–Worst One-Slide-per-Patient AUC After Tumor-Content Filtering**					
**Task**	**Analysis Cohort**	**WSI Passing Filter**	**Best-Case AUC**	**Worst-Case AUC**	**Best–Worst ΔAUC**
ODX	No tumor-content filter	400	0.972	0.626	0.346
ODX	Excluding tumor-negative/insufficient-QC WSIs	388	0.972	0.642	0.330
ODX	Excluding low-tumor-content WSIs	358	0.969	0.661	0.308
HER2	No tumor-content filter	400	0.896	0.530	0.366
HER2	Excluding tumor-negative/insufficient-QC WSIs	388	0.896	0.578	0.318
HER2	Excluding low-tumor-content WSIs	358	0.896	0.629	0.267

**Table 5:** Calibration under random one-slide-per-patient inference and patient-level multi-WSI aggregation. Fold-model probabilities were averaged before calculating the displayed patient-level Brier scores. Lower values indicate better probabilistic accuracy. Differences are calculated as aggregated minus random values; negative values favor aggregation.

Task	Model	Random Brier	Aggregated Brier	ΔBrier
ODX surrogate	DTFT	0.141	0.121	−0.020
	ABMIL	0.138	0.119	−0.019
	S4Model	0.146	0.130	−0.016
	DAMLN	0.187	0.161	−0.026
	RRTMIL	0.172	0.162	−0.010
	TransMIL	0.176	0.148	−0.028
HER2	DTFT	0.183	0.169	−0.014
	ABMIL	0.161	0.152	−0.009
	S4Model	0.280	0.174	−0.106
	DAMLN	0.292	0.253	−0.039
	RRTMIL	0.176	0.159	−0.017
	TransMIL	0.166	0.145	−0.021

## Data Availability

The TCGA-BRCA and CPTAC-BRCA molecular data used in this study are available through the Genomic Data Commons and CPTAC data resources, and associated public pathology images are available through their linked repositories. Fold definitions, slide lists, derived labels, model code, and analysis scripts are available from the corresponding author upon reasonable request and will be released in a public repository with the final published version.

## Abbreviations

AI	Artificial intelligence
AUC	Area under the receiver operating characteristic curve
CPTAC	Clinical Proteomic Tumor Analysis Consortium
FFPE	Formalin-fixed, paraffin-embedded
H&E	Hematoxylin and eosin
HER2	Human epidermal growth factor receptor 2
HR	Hormone receptor
HSV	Hue, saturation, and value
MIL	Multiple-instance learning
ODX	Research-derived recurrence-score surrogate
PPV	Positive predictive value
NPV	Negative predictive value
QC	Quality control
RNA	Ribonucleic acid
TCGA	The Cancer Genome Atlas
WSI	Whole-slide image
